# Autologous reconstitution of human cancer and immune system *in vivo*

**DOI:** 10.18632/oncotarget.14026

**Published:** 2016-12-19

**Authors:** Juan Fu, Rupashree Sen, David L. Masica, Rachel Karchin, Drew Pardoll, Vonn Walter, D. Neil Hayes, Christine H. Chung, Young J. Kim

**Affiliations:** ^1^ Department of Otolaryngology - Head & Neck Surgery, SKCCC, Johns Hopkins Hospital, Baltimore, MD, USA; ^2^ Department of Biomedical Engineering and The Institute for Computational Medicine, SKCCC, Johns Hopkins Hospital, Baltimore, MD, USA; ^3^ Bloomberg-Kimmel Institute for Cancer Immunotherapy, SKCCC, Johns Hopkins Hospital, Baltimore, MD, USA; ^4^ Department of Biochemistry and Molecular Biology, Penn State Milton S. Hershey Medical Center, Hershey, PA, USA; ^5^ UNC Chapel Hill School of Medicine, Lineberger Comprehensive Cancer Center, Chapel Hill, NC, USA; ^6^ Department of Head & Neck - Endocrine Oncology, Moffitt Cancer Center, Tampa, FL, USA; ^7^ Department of Otolaryngology - Head & Neck Surgery, VICC, Vanderbilt University Medical Center, Nashville, TN, USA

**Keywords:** tumor microenvironment, autologous reconstitution, STAT3, humanized mice, head neck carcinoma, Immunology and Microbiology Section, Immune response, Immunity

## Abstract

Correlative studies from checkpoint inhibitor trials have indicated that better understanding of human leukocytic trafficking into the human tumor microenvironment can expedite the translation of future immune-oncologic agents. In order to directly characterize signaling pathways that can regulate human leukocytic trafficking into the tumor, we have developed a completely autologous xenotransplantation method to reconstitute the human tumor immune microenvironment in vivo. We were able to genetically mark the engrafted CD34+ bone marrow cells as well as the tumor cells, and follow the endogenous leukocytic infiltration into the autologous tumor. To investigate human tumor intrinsic factors that can potentially regulate the immune cells in our system, we silenced STAT3 signaling in the tumor compartment. As expected, STAT3 signaling suppression in the tumor compartment in these autologously reconstituted humanized mice showed increased tumor infiltrating lymphocytes and reduction of arginase-1 in the stroma, which were associated with slower tumor growth rate. We also used this novel system to characterize human myeloid suppressor cells as well as to screen novel agents that can alter endogenous leukocytic infiltration into the tumor. Taken together, we present a valuable method to study individualized human tumor microenvironments in vivo without confounding allogeneic responses.

## INTRODUCTION

Durable clinical responses with immune checkpoint inhibitors recently have prompted an expansion of clinical trials with various combinations of immunomodulators for cancer patients [1, 2]. Clinical trials are frequently driven by correlative biomarker analyses that attempt to predict clinical responses based on expression of certain molecules in the human tumor microenvironment [3]. While these studies can yield potential discovery panels and possible selection criteria for the subsequent clinical trials, they provide a retrospective “snapshot” correlations and thus an incomplete mechanistic picture of the dynamic human tumor microenvironment. For mechanistic evaluations, preclinical testing of immunotherapeutic agents relies upon syngeneic immunocompetent murine models or genetically engineered mouse models (GEMM), which are limited by their inadequate genetic and biological representation of corresponding human cancers [4, 5]. Patient derived xenotransplant (PDX) models that introduce human tumor xenografts into immunodeficient mice that are reconstituted with human hematopoietic cells [6–8] are limited by the fact that the adaptive human immune system developing in these mice is allogeneic to the tumor, even when the human thymus is concurrently implanted or when human HLA transgenic mice are used as recipients [9–11]. Thus, anti-tumor responses are dominated by alloantigen rather than tumor-specific antigen recognition.

A method that can simultaneously reconstitute autologous human tumor and human immune cell in vivo would allow important immunological studies to expedite translation of immunotherapeutic trials. To partly address this problem, autologous T-cells have been adoptively transferred into a tumor bearing mice, but this exogenous method failed to account for other important non-lymphocytic hematopoietic cells [12]. Others are currently generating murine lines with the different combinations of human HLA to overcome this allogeneic response but the polymorphism of HLA genes makes it virtually impossible to match all HLA alleles with a given tumor or transplanted immune system [13–15].

We describe here a system to reconstitute a fully autologous human tumor microenvironment that would allow direct studies of molecular pathways that can drive the relationship between tumor cells and the various components of hematopoietic cells. We use this system to analyze the immunological consequences of STAT3 signaling disruption on the human tumor microenvironment (TME). We demonstrate that genetic silencing of STAT3 in the human tumor compartment can mediate enhanced infiltration of human T cells into the developing human tumor in vivo. In addition, we used this novel system to characterize human myeloid suppressor cells, as well as to screen novel agents that can alter endogenous leukocytic infiltration into the tumor.

## RESULTS

### Autologous reconstitution of human tumors in vivo

**Figure 1:**
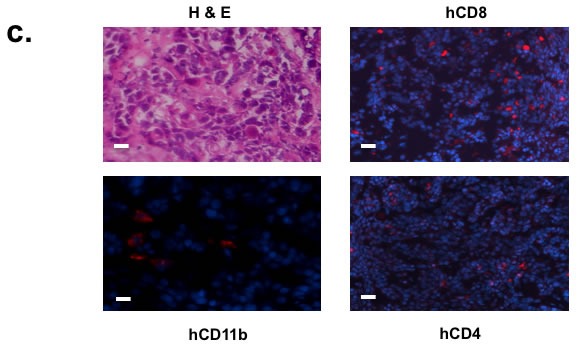
Human squamous cell carcinoma can be xenotransplanted into humanized mice autologously **a.** Schematics of autologous reconstitution of HNSCC. **b.** After engraftment with CD34+ bone marrow cells from HNSCC patient undergoing surgery, humanized mice showed presence of human lymphocytic and myeloid cells (CD11b+), including quantifiable human NK cells (NKG2D+NKp46+) in the spleen. Gated mononuclear cells were plotted for human CD45 and murine CD45 levels (upper panels). Gated human CD45 then were plotted for human CD markers as noted in the axis (lower panels). In brief, peripheral blood was stained with murine CD45, human CD45, and **c.** Surgical tumor specimens from the same patient whose CD34+ BMC were used to engraft NOG-A2 mice (from 1b) were implanted into the same humanized mice. The engrafted tumor was harvested and stained for tumor infiltrating human CD8, CD4, and CD11b cells. The primary antibodies for these stainings were rabbit anti-human CD8, rat anti-human CD4, and rabbit anti-human CD11b conjugated to Alexa Fluor® 568. The bar scale is 100µ. Upper left panel is an H&E stain of the same tumor.

We initially humanized NOD/SCID/γcnull HLA-A2+ (NOG-A2 crossed to human HLA-A0201 transgenic mice) mice that we generated with CD34+ enriched cells from cord blood from HLA-A2+ C-section specimens. These humanized NOG-A2 mice were found to repopulate both human lymphoid and myeloid hematopoietic cells (Supplemental Figure 1a). HLA-A2+ HNSCC tumors were initially xenotransplanted into these humanized mice to reconstitute the human HNSCC allogeneically, and we found that human T-cells can infiltrate into the tumor microenvironment (Supplemental Figure 1b). To bypass the allogeneic response in this system, we developed a completely autologous human tumor/hematopoietic system using HNSCC surgical specimens and bone marrow cells (BMC) harvested during surgery. In brief, BMC and HNSCC tumor from HLA-A2+ patients undergoing composite surgical resection of HNSCC with reconstruction of the mandible with fibula bone were harvested. Primary tumor and CD34+ BMC from the fibula bone were initially engrafted into NOG-A2 separately (Figure 1a). In the bone marrow engrafted mice, harvested hematopoietic cells in the spleen showed mature human lymphocytic and myeloid cells (Figure 1b). Tumors derived from the same patient as the donor BMC, which had been passaged in parallel to non-humanized NOG-A2 mice, were then transplanted into the humanized mice to reconstitute a fully autologous tumor microenvironment (TME). When the transplanted tumor was analyzed, both human lymphoid and myeloid cells were found to infiltrate into the TME (Figure 1c).

Typically, the chimerism of the hematopoietic system rates ranged from 70-90% as defined by human CD45 level with respect to total mononuclear cell from the spleen asmurine CD45 from the peripheral blood as shown in Figure 1b. Splenocytes also contained appropriate levels of human T-cells (CD4 and CD8), myeloid cells (CD11b and CD11c), and NK cells (NKG2D, NKp46). Poor engraftment was defined by global fragility of the mice in 2-86 weeks post transfer of BMC, and only healthy mice after 2 months were implanted with human tumor tissues. After engrafting approximately 110 mice with this method from 12 donors, 55% of the mice survived beyond 6 weeks as shown in Supplemental Figure 2. It should be noted that many of these mice that did not survive beyond 2 months received BMC from patients aged 70 or above, and we have stopped using fibula BMC from those over 65 years old. With this optimization, all the mice survived to 2 months for tumor injection. While engraftments with cord blood was limited by the available abundance of CD34+ cells (104-5 per mouse), the greater abundance of fibular BMC allowed the transfer of 2 x105 CD34+ cells (per mouse) to allow comparable engraftment rates as with cord blood [16]. Unlike the implantation of PDX into non-humanized mice, most, if not all, of the mice had human HNSCC tissue growing in subcutaneous tissue for our analysis. Therefore, the “rate-limiting step” of this autologous reconstitution was the engraftment of the BMC step. Limited sources of patients undergoing fibular resections prevented titration of BMC engraftments on a per cell basis, but our global engraftment rates compared favorably with cord blood engraftments [16].

**Figure 2:**
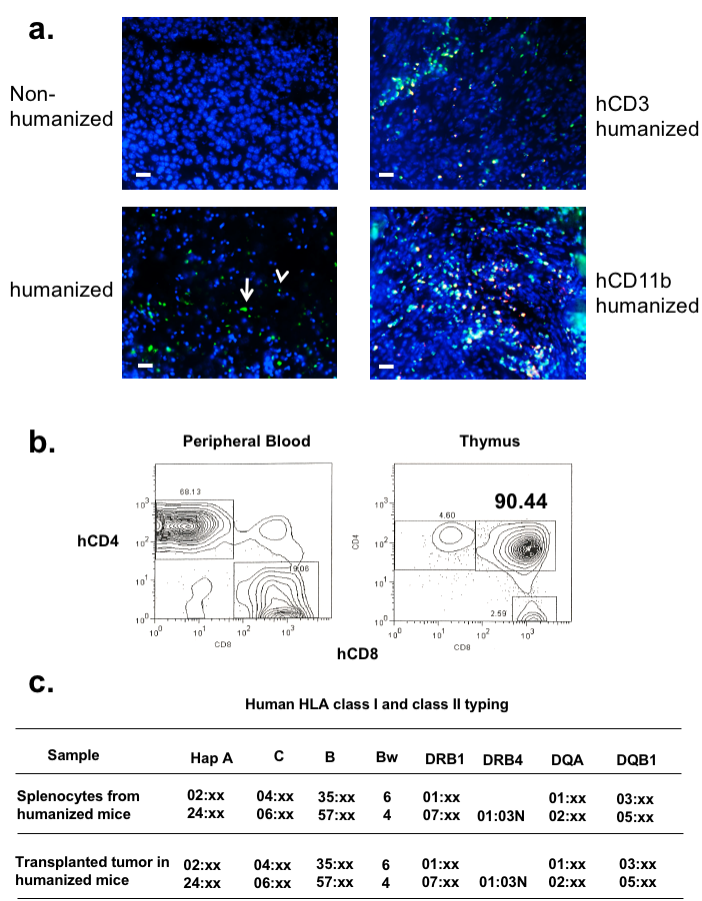
Autologously reconstituted human carcinoma is infiltrated with endogenous human hematopoietic lymphocytes that mature in the thymus **a.** CD34+ BMC were transduced with lentivirus vectors containing GFP protein prior to BMC engraftment. Autologously reconstituted tumor was harvested, stained with DAPI, and immunofluorescent microscopy was performed. The left panels show tumor with only DAPI counterstain. Left upper panel is tumor xenografted into non-humanized mice, while the left lower panel is the tumor in autologously humanized mice (white arrowhead – lymphocyte; white arrow – myeloid cells). Slides were also co-stained with human CD3 and CD11b conjugates (right panels). The bar scale is 100µm. **b.** Human lymphocytes from humanized NOG-A2 (HLA-A0201 transgene) mice mature in the thymus. RBC depleted peripheral blood (PBL) from humanized mice were stained with hCD4 and hCD8. Thymus from these humanized mice were harvested and stained for hCD4 and hCD8. **c.** After tumors grew to 1 cm3 in autologously humanized mice, splenocytes and tumor were harvested for human HLA class I and class II typing. CD3 depletions were performed on the tumor specimens prior to this analysis. The haplotype report showed that human HLA class I and class II types between the tumor and the immune cells were identical. .

In order to ensure that the tumor infiltrating human T-cells are derived from the engrafted hematopoietic cells and not from the tumor infiltrating lymphocytes (TIL) from the tumor tissue, we labeled the human CD34+ BMC with lentiviral vectors that expressed GFP proteins. Once the engrafted lymphocyte progenitors were found to produce GFP positive lymphocytes in the peripheral blood (data not shown), autologous tumor was implanted, and the immunofluorescent microscopy clearly showed GFP+ immune cells in the TME with both lymphocytic and myeloid histology (Figure 2a - left lower panel). Immunohistochemical staining with human CD3 and CD11b conjugates co-localized to these GFP positive cells and confirmed the presence of both human lymphoid and myeloid cells in the TME (Figure 2a - right panels). While these experiments demonstrated that a significant proportion of T cells in the TME are derived from the engrafted BMC, they do not formally distinguish what proportion are from homeostatic expansion of mature T cells in the bone marrow vs. differentiation of CD34+ progenitor cells. When we probed the thymus from these mice, thymocytes from these engrafted mice were predominantly double positive human T-cells, suggesting that de novo development of human T cells in the murine thymus with human HLA-A2 expression was indeed operative (Figure 2b). Since we showed that human BMC-derived cells from CD34+ grafts indeed reconstituted the murine thymic stroma, we reasoned that these T cells should be tolerant to the HLA-A2+ transplanted tumor that is seen in human tumor [17]. Lastly, we haplotyped the transplanted tumor and the human leukocytes from peripheral blood of these autologously humanized mice to validate that the HLA type was a complete match (Figure 2c).

### Humanized mice can generate CD4 dependent humoral responses

**Figure 3:**
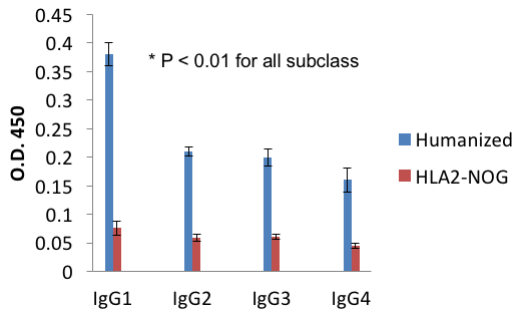
Autologously xenotransplanted mice can mount a T-cell dependent antibody response *in vivo* After vaccination and boost with DHP-KLH, serum levels of DNP specific human IgG1-4 in DNP-KLH immunized mice were measured using ELISA in triplicate samples. All these representative figures are from independent experiments performed from three tumor bearing humanized and three non-tumor bearing non-humanized mice (NOG-A2).

In order to assess the function of the adaptive immune system in these humanized NOG-A2 mice, they were vaccinated with KHL antigen and their CD4 dependent generation of antibodies against KHL analyzed (Figure 3) [18]. Following immunization and 2 week booster with NDP23-KLH, serum levels of DNP specific human antibodies were quantitated with ELISA. These humanized mice generated increased IgG responses to all IgG subtypes, demonstrating that these mice can generate CD4 dependent humoral responses in vivo.

### STAT3 can mediate pro-carcinogenic human T-cell infiltration into human tumor to increase the growth rate in vivo

**Figure 4:**
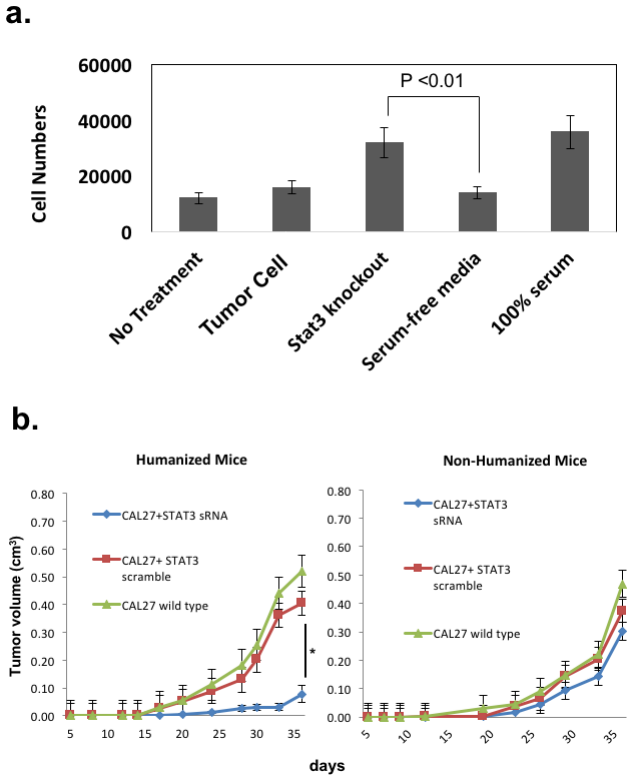
STAT3 signaling in the human tumor cells can mediate human T-cell trafficking into the tumor *in vivo* to increase the tumor growth rate in an allogeneic reconstitution system **a.** Lymphocytes from HNSCC patients undergoing surgery were harvested and assayed for migration towards conditioned supernatants from Cal27 tumor cell lines in this chemotaxis assay (5 experiments performed in triplicates) [19]. Negative controls consisted of media with no exposure to tumor cells (“no treatment”), and 100% serum as positive controls. Cal27 cells were transduced with scrambled lentivirus (“tumor cells”) or STAT3 siRNA lentivirus (“Stat3 knockout”). **b.** HLA-A2+ Cal27 HNSCC cell lines were transduced with either scrambled siRNA or STAT3 siRNA, and these tumor cells were injected into humanized mice (7-10 mice/group). Growth rates were followed after xenotransplantation (left panel, *P<0.01). These tumor cells were also injected into non-humanized NOG-A2 mice (right panel). These experiments were independently repeated 3 times. **c.** Harvested tumors were stained with H&E and analyzed. The bar scale is 100µ. **d.** Tumor transduced with STAT3 siRNA showed greater number of tumor infiltrating human CD45+ cells (left panel). Harvested tumor from the two groups of tumor from humanized mice (scrambled siRNA control and siRNA treated Cal27) were stained with human CD45-Alexa Fluor 568 conjugate and CD45+cells were counted over 10 fields and averaged. We also ensured that transduced tumor cells had stable suppression of STAT3 at the time of tumor harvest (right panel) by performing pSTAT3 expression analysis in the two groups of tumors from Fig. 4b at the time of tumor harvest on day 36. The bar scale is 100µ.

We previously found that STAT3 signaling in tumor cells can potentially decrease leukocyte migration in vitro [19]. When we specifically tested the migratory behavior of CD8+ T-cells from HNSCC cancer patients, we noted that STAT3 signaling in tumor cells can suppress lymphocytic migration in vitro. Conditioned media from HNSCC tumor cells did not induce T-cell migration, while media from same cell lines whose STAT3 gene was suppressed (“Stat3 knockout”) induced T-cell migration as much as our positive control treatment (“100% serum”)(Figure 4a). We initially probed the TCGA for STAT3 gene expression and phospho-STAT3 levels in our banked specimens (data not shown). However, while analyzing the transcript levels in the public cancer database such as TCGA and archived specimens may provide correlative support, [20], such methods cannot distinguish STAT3 signaling in the tumor cells from the tumor infiltrating immune cells. With our ability to reconstitute the tumor tissue from the tumor cells and immune cells, we reasoned that our method can directly study lymphocytic trafficking mediated by tumor intrinsic STAT3 signaling in vivo.

We initially started with an allogeneic HLA-A2+ HNSCC line (Cal27) transplanted into CD34+ BMC reconstituted NOG-A2 mice. Using STAT3 siRNA method, we silenced STAT3 signaling in Cal27 tumor cells and compared them with Cal27 transduced with control lentivirus. Despite the pleiotropic effect of STAT3, STAT3 silencing did not affect its growth rate in vivo when transplanted into an immunodeficient non-humanized NOG-A2 mice (Figure 4b - right panel). This demonstrated that STAT3 signaling in the tumor cells did not affect the immune cell independent apoptotic rate of the tumor in our system. However, in the context of a humanized mice, STAT3 silencing significantly decreased the growth rate of the reconstituted tumor (Figure 4b - left panel). When we compared the histology between the STAT3 silenced and control tumors, we noted increased tumor necrosis in the STAT3 silenced tissues (Figure 4c). When we quantitated the TILs using human CD45-fluorophore conjugate in IHC, STAT3 suppressed tumor cells showed increased tumor infiltrating human CD45 cells (Figure 4d - left panel). We confirmed that STAT3 expression was stably silenced after the tumors were harvested at the end of the in vivo experiments (Figure 4d - right panel).

### STAT3 can mediate pro-carcinogenic human immune cell infiltration into autologous human tumor to increase the growth rate in vivo

**Figure 5:**
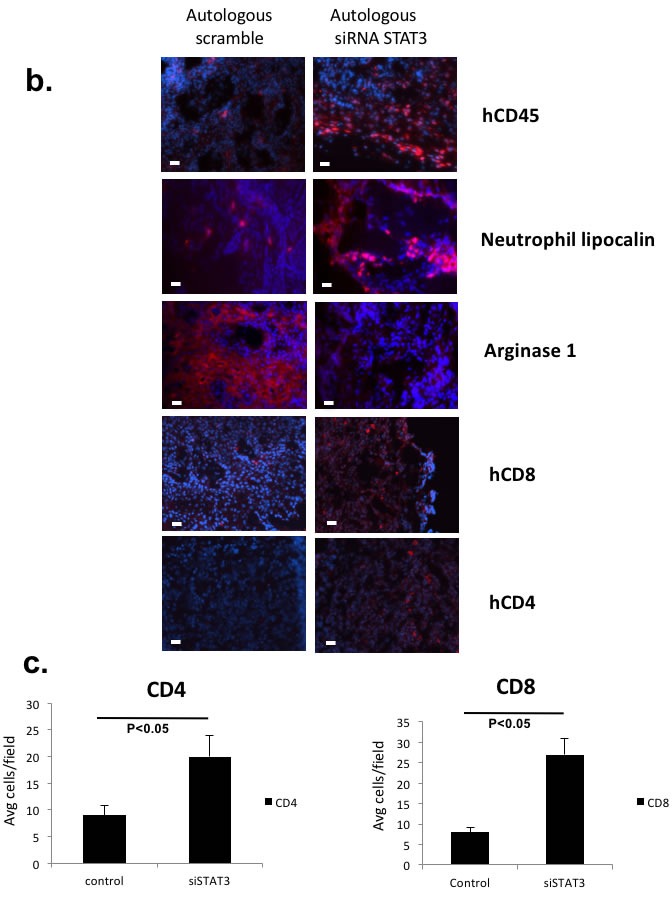
Tumor intrinsic STAT3 signaling that can regulate human leukocytic infiltration and immunosuppression can direct tumor growth *in vivo* in an autologous system **a.** Patient derived tumors were transduced with siRNA lentivirus (scrambled or STAT3 siRNA) and autologously implanted into humanized mice and non-humanized mice. Shown are autologous reconstitution from 4 donors. Autologously reconstituted HNSCC tumor with STAT3 suppression had decreased tumor growth rate (center panels) while the matched PDX in non-humanized mice demonstrated no differential tumor growth rates (left panels). Four experiments were performed with 4-5 mice per group. Harvested tumor from each group had persistant STAT3 suppression at the end of the experiment (right panels). For some xenoplants, the tumor did not engraft as demonstrated, particularly in non-humanized mice (autolog 3, 4). (Red – scrambled siRNA, Blue – STAT3 siRNA, Green – untreated tumor)(**, p<0.01; *, p<0.05). **b.** Tumor STAT3 signaling altered the infiltrating immune cells as well as the metabolic milieu of the TME. Harvested tumor were stained with human leukocyte markers and human arginase-1 antibodies with immunofluorescence. **c.** For hCD4 and hCD8 staining, positive cells were quantitated in 10 randomly selected different fields at 40x magnification. The bar scale is 100µ. STAT3 suppressed tumor tissue showed increased tumor infiltrating human CD4 and CD8 T-cells..

Since allo-antigen immune responses in the TME can confound the tumor growth rate in this allogeneic system, we tested the STAT3 signaling in primary HNSCC tumors using our autologous HNSCC tumor engraftment system. We silenced STAT3 signaling by transducing homogenized tumor cells between the serial passages in the non-humanized mice prior to the autologous reconstitution in the humanized mice. To ensure STAT3 suppression in our system did not affect the apoptotic pathway, we injected these matched xenotransplants into non-humanized NOG-A2 mice and found no differential growth rates between the control scrambled siRNA vs STAT3 siRNA transduced tumors (Figure 5a - left). These matched tumors were then injected into mice humanized from BMC from the same cancer patient. In 3 of 4 human HNSCC specimen tested, STAT3 silencing significantly decreased the growth rate of autologously reconstituted tumor (Figure 5a - center). We harvested the tumors from these experiments and probed for pSTAT3 signaling, and noted persistent STAT3 suppression at the end of 35 days in the STAT3 siRNA treated mice (Figure 5a - right). In some of the experiments, we noted no tumor engraftment in the non-humanized mice. As shown in these autologous experiments, we did note variability in the tumor growth rates. For those mice that failed to grow tumors, we could not analyze their STAT3 suppression levels.

In mice with appropriate tumor engraftment, we probed the TME for leukocyte infiltrations between the STAT3 suppressed group and the control group (Figure 5b and 5c). In the STAT3 suppressed reconstitution, both myeloid and lymphoid cells were noted to be increased. We also stained the tissue for human arginase-1 that is transcriptionally upregulated with STAT3 [21], and we found dramatic reduction of this immunosuppressive enzyme in the STAT3 suppressed tumor. In brief, our autologous system has allowed the direct testing of tumor endogenous factors that can mediate not only the tumor trafficking of human immune cells, but also the metabolic microenvironment of the tumor tissue.

### Autologously reconstituted human tumor can be used to study immunosuppressive myeloid cells

**Figure 6:**
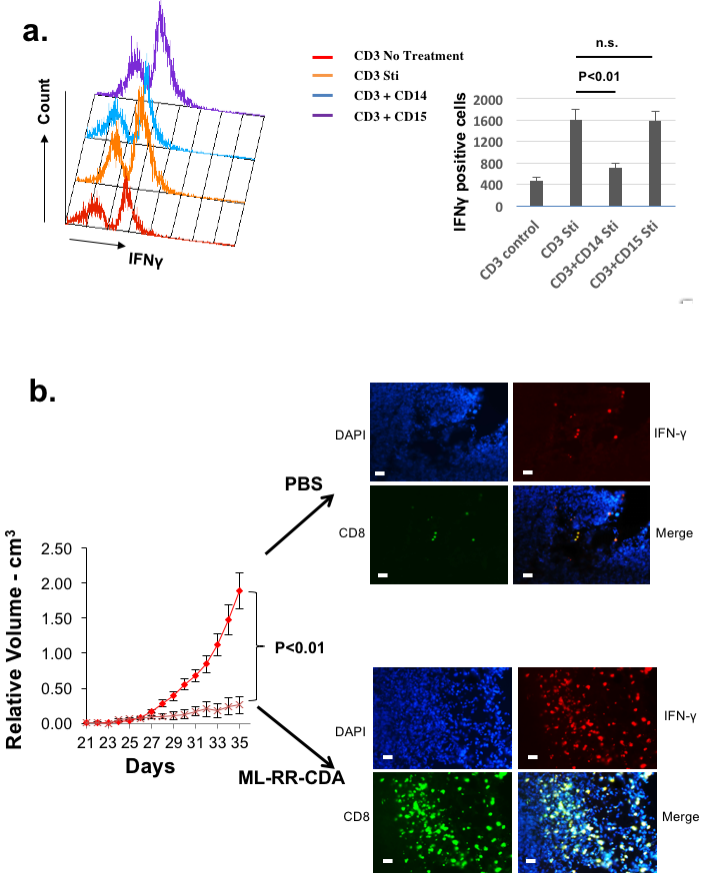
Autologously reconstituted HNSCC tumor system can be used to study human myeloid derived suppressor cells as well as novel immunomodulatory agents **a.** Splenocytes from autologously reconstituted human tumor bearing mice were harvested, and CD11b+CD14+DRlow (CD14) and CD11b+CD15+DRlow (CD15) cells were sorted to homogeneity to ensure monocytic and granulocytic histology (data not shown)[21]. Human CD3+ cells were co-cultured with CD11b+CD14+HLA-DRlow and CD11b+CD15+HLA-DRlow cells in complete RPMI media and stimulated (Sti) with PMA/Ionomycin. CD3+IFNγ+ cells were quantitated with flow cytometry as the measurement of T-cell suppression when mixed with MDSC. **b.** Palpable tumors of equivalent size in both groups were treated intratumorally with either PBS control or ML-RR-CDA at 20µg per injection and the tumor growth was followed. Experiment repeated twice with 4-5 mice per group. At the completion of experiment, the tumor was harvested and stained with human CD8, IFNγ, and DAPI counterstain (right panels). The bar scale is 100µ.

While our previous studies were focused on the T-cells, we inquired whether the myeloid population can also be studied in our model. Since human HNSCC can expand the MDSC population, we harvested the spleen in these autologous human tumor bearing humanized mice and sorted human MDSC as described before and tested their ability suppress human T-cells. Our previous work with murine myeloid suppressor cells suggested that the monocytic MDSC represent the more potent T-cell suppressor [22], so we hypothesized that the human MDSC may have similar activity. We sorted CD11b+CD14+DRlow as well as CD11b+CD15+DRlow from these mice and compared their T cell suppression activity. Consistent with our previous work in the murine system, we found that the monocytic CD14+ MDSC from these human tumor bearing humanized mice can suppress human T-cell activation ex vivo while the granulocytic CD15+MDSC failed to demonstrate this (Figure 6a) [22]. We titrated these MDSC with different ratios of human T-cells, and their suppressive activity was comparable to the MDSC from HNSCC patients (Supplemental Figure 2).

### Humanized mice reconstituted with autologous human tumor can be used to assess the activity of intra-tumoral injection of novel immunomodulatory agents

As a scalable in vivo system, our method is also amenable for testing immunotherapeutic agents in a late preclinical phase as well as in parallel to clinical trials. To test our system for novel immunotherapeutic adjuvants, we treated these autologously reconstituted human tumors with intra-tumoral synthetic cyclic dinucleotide, ML-RR-CDA, currently being tested in a Novartis phase I clinical trial [23]. Compared to PBS controls, intra-tumoral ML-RR-CDA induced a potent Th1 (IFNγ) response in the tumor microenvironment that resulted in an in vivo anti-tumor response of the reconstituted human HNSCC tumor (Figure 6b). These results were comparable to the efficacy of ML-RR-CDA in murine tumor models tested previously [24].

## DISCUSSION

The translational limitations of genetically engineered murine models were recently reviewed [25]. While patient derived xenograft system in immunodeficient mice may recapitulate some of the genetic diversity and heterogeneity of human tumors for personalized cancer research of signaling pathways of the tumor cells, it cannot be used to study immunotherapeutic modalities that are now establishing itself as an important aspect of clinical oncology in most forms of human cancer [26–28]. Genetically engineered models also have limitations for preclinical testing of immunomodulating agents since some of the phenotypes derived from genetic manipulations may minimize the stromal and the immunological context of the autochthonous tumor [25]. Although xenografts into humanized models are being used to study the immunological responses to the transplanted tumor, an important limitation is the unavoidable allogeneic responses that may confound the anti-tumor responses seen in these mice. Autologous adoptive transfer of T cells was recently demonstrated, but this method does not allow for endogenous immune infiltration into the developing tumor [12]. Our autologous system alleviates these problems and provides a method to study endogenous lymphocytic and myeloid cell trafficking into the human tumor in vivo.

Using our autologous model, we were able to genetically modify STAT3 signaling in the tumor compartment and demonstrate its ability to regulate the tumor infiltrating human lymphocytes that can promote the tumor growth rate [19, 29]. We also demonstrated our ability to genetically modify the human hematopoietic cells, so we now have the capability of studying the human tumor-immune cells relationships in vivo, both in an allogeneic and an autologous system (Figure 2A). Since this system is completely autologous, any observed in vivo effect can be attributed to the experimental interventions rather than the allogeneic response that is inherent in the current PDX systems. Moreover, rather than relying on correlative relationships from specimens, we can now directly test specific immune-oncologic signaling pathways in the human TME in vivo to complement mechanistic studies on genetically engineered murine tumor systems [42]. Furthermore, our autologous platform also allows future study of human T-cells against tumor neoantigen development in a preclinical setting with the recent advances in TCR sequencing technology [30]. Lastly, our method can also be used to develop rare tumor models that do not have syngeneic murine models for preclinical developments of novel immune-oncologic agents.

In multiple series of immune checkpoint blockade trials, there has been a consistent demonstration that enhanced lymphocytic infiltration has been associated with improved clinical responses [31–33]. Towards the goal of targeting the adaptive immune resistance mechanism of the developing tumor, we and other authors previously noted that tumor cell STAT3 signaling can potentially regulate the leukocytic infiltration into the TME as part of its pleiotropic carcinogenic effects in murine models [19, 34, 35]. To the extent that STAT3 signaling mediates immunomodulatory effect within the TME, our experiments support future STAT3 clinical trials that combine immune checkpoint inhibitors with inhibitors of STAT3 signaling [34, 36–38]. In fact, a recent study has reported promising clinical results with STAT3 inhibition [39].

While our reconstitution system has yet to account for the mesenchymal cells of the tumor microenvironment, there are several biomedical implications from our autologous reconstitution of the human tumor in vivo. Since bone marrow aspiration for autologous xenotransplantation is clinically feasible with minimal risks at the time of surgery, routine autologous reconstitution of an individual tumor in a humanized system is now possible. This system substantially increases the personalization of cancer treatment whereby immune-oncologic treatment of an individual tumor can be studied in the context of their own tumor microenvironment. These methods also allow the screening and testing of immunotherapeutic agents that have gained prominence since the introduction of immune checkpoint inhibitors into the clinic [40, 41].

## MATERIALS AND METHODS

### Reagents, cells, mice

Antibodies against anti-human CD3, CD4, CD8, CD11c, CD11b, CD14, CD15, CD16, CD19, CD33, CD34, CD45, CD56, NKP46, CD123, arginase-1, neutrophil lipocalin, IFNγ, HLA-DR, and pStat3 were purchased from BD Biosciences, Biolegend, or ebiosciences. Synthetic 2’-5’ linked [R,R] dithio cyclic di-AMP (ML-RR-CDA) was a gift from Aduro Biotech. NOD.scid.IL2Rγcnull (NOG) mice were crossed to NOD.HLA-A2*0201 transgenic mice (Jackson) to generate NOD/SCID/γcnull HLA-A2+ (NOG-A2) mice in our lab and housed according to JHH Animal Care. In some cases, HSCFTL mice [NOD-Cg-PrKdcscidIL2γgtm1sug/JicTac mice engrafted with human CD34+ hematopoietic stem cells (HSCs) (Taconic), NSG, or NSG-HLA-A2.1 mice (Jackson) were used. Human CD34 microbead kit and pan T-cell isolation kit were purchased from Miltenyi Biotec. Human HNSCC cell lines used consisted of Cal27 and SCC25 (ATCC). The cell lines were authenticated by a short tandem repeat profiling analysis using the AmpFISTR Identifier PCR Amplification kit (Applied Biosystems) at the Genetic Resources Core Facility, JHH [43]. Cells were maintained in DMEM medium typically supplemented with 10% FBS and penicillin (100 U/mL) and streptomycin (100 mg/mL) and maintained in a humidified incubator at 37°C in a 5% CO_2_ atmosphere.

### TCGA transcriptome analysis

STAT3 gene expression levels (n = 279) were obtained from The Cancer Genome Atlas dataset based on methods described in the Supplementary Materials [20]. A two-sided Wilcoxon rank sum test was used to assess the difference in the expression levels between the mesenchymal and classical subtypes.

### TCGA RPPA analysis

Phosphorylated STAT3 expression levels (n = 200) were obtained from The Cancer Genome Atlas dataset. Briefly, the protein lysates from frozen tumors were prepared and analyzed by reverse phase protein array (RPPA) as previously published [44, 45]. The primary antibody, phospho-STAT3-Tyr705 (Cell Signaling Technology), was stained at 1:500 dilutions. The difference in the expression levels between the mesenchymal and classical subtypes was assessed using a two-sided Wilcoxon rank sum test.

### Engraftment of hematopoietic stem cells

8-10 weeks NOG-A2, NOG, NSG, or NSG-HLA-A2.1 mice were lethally irradiated with 4-5 Gray prior to tail vein injection with human umbilical cord blood cells (CBC) or human bone marrow CD34+ hematopoietic stem cells after 1:2 dilution with PBS/1%FBS and passed cells through 100µm cell strainer (Corning). Typically, the CBC and bone marrow cells (BMC) were fractionated with density-gradient Ficoll-Hypaque. The buffy coat layer cells were washed twice with PBS/1% FBS, and CD34+ cells were positively selected with Miltenyi column. 5×104-2×105 CD34+ cells in 500µl PBS were used to humanize lethally irradiated mice. In some cases, these CD34+ BMC were transduced with high titer GFP lentivirus prior to transfer. Peripheral blood or spleen were analyzed for engraftment of human hematopoietic cells after 6 weeks.

### Autologous and allogeneic reconstitution of tumor

For autologous reconstitution, tumor specimen and fibular bone marrow cells from HLA-A2+ patients undergoing head and neck cancer surgery were harvested. BMC were used to engraft NOG-A2 mice as noted above. In parallel to this engraftment, the tumor tissue (approximately 0.125cm3) from the same patient were cut and digested in media containing DNAse I (Roche) and Liberase Blendzyme 2 (20,000 Mandl U/ml) (Roche) 20 minutes at room temperature. The digested tumor tissue was passed through 100µm cell strainer and washed with PBS/1%FBS. 1-2×106 tumor cells mixed with BD Matrigel TM basement membrane matrix (BD Bioscience) in 100µl PBS. These cells were subcutaneously injected initially into non-humanized NOG-A2 mice. Once the mice engrafted with their matched BMC were confirmed to have CD4+ and CD8+ T-cells in the peripheral blood, the matched tumor tissue was implanted subcutaneously into the bone marrow engrafted humanized NOG-A2 mice. In some cases, the tumor cells were transduced with STAT3 or control siRNA lentivirus prior to xenotransplantation. For allogeneic reconstitution, HLA-A2+ tumor cell lines were injected subcutaneously into humanized NOG-A2 mice from allogeneic BMC or CBC.

### STAT3 silencing with lentivirus and editing via CRISPR

Transfections of tumor cells (both primary and tumor cell lines) with STAT3 small interfering RNA (siRNA) oligonucleotide (Santa Cruz) were conducted using LipofectAMINE 2000 (Invitrogen Life Technologies). For control samples, cells were transfected with scrambled small interfering RNA oligonucleotide or LipofectAMINE alone. The cells were transfected with 15nM of STAT3 or scrambled siRNA and cultured for two days. Mission TRC-Hs (Sigma) clone sets of sequence-verified siRNA lentiviral plasmids were obtained from the JHU High Throughput Biology Center. VSV-G pseudo-typed virus was produced by the Johns Hopkins Neurosurgery Vector Core by co-transfecting 293T cells with an siRNA transducing vector and two packaging vectors: psPAX2 and PMD2.G [19]. In some cases, STAT3 was edited using CRISPR/CAS9 kit was purchased from Origene (KN204922) containing GFP reporter gene. Human Cal27 cells were seeded to reach 50-70% confluency day a before transfection. The scramble control DNA and STAT3 gRNA vector with donor DNA were transfected into Cal27 cells separately. After 2-5 passages the GFP positive cells were sorted. Additionally, GFP+ cells were selected with puromycin 1-2µg/ml for purity, whereas western blots were applied to confirmed complete STAT3 knockout or silencing in transfected cells.

### Tumor treatment assay

Humanized mice were subcutaneously injected with 106 Cal27 human squamous cells or serially passaged patient derived tumor tissue. In some series, the tumor cells were infected with lentivirus as noted above for STAT3 silencing/editing. Once palpable tumors developed (20-30 days), 20µg ML-RR-CDA (in 100µl PBS)(Aduro) was injected intra-tumorally twice over 1 week period in therapeutic experiments with adjuvants. The control groups were injected with PBS alone. For all these experiments, 3-5 mice were used per group. Palpable tumor measurements were initiated once all 3 dimensions reached anywhere from 2-4 mm. Relative tumor volume V was calculated using the formula V = (W2 X L)/2, where L is the longest and W is the shortest diameter. Tumor growth was monitored every alternate day and animals were euthanized when tumors reached 15-20 mm in diameter.

### Flow cytometry

Peripheral blood and spleen was collected from humanized mice. The white blood cells were isolated using Ficoll-paqueTM plus (GE Healthcare) in a ratio of 2:1. CD4, CD8, CD3, CD45, CD11b, CD11c, CD56, CD16, NKP46, CD123, CD33, HLA-DR staining were determined by flow cytometry (FACSCalibur) and analyzed with FloJo software (Tree Star). For pStat3 (pY705) staining, tumor was harvested and CD45 depleted tumor cells were fixed in 2% paraformaldehyde/PBS for 10 min at 37°C and permeabilized by resuspending with vigorous vortexing in 500 µl ice-cold 90% methanol per 106 cells and incubated at 4°C for 30 min on ice. After PBS washing and resuspension, cells were stained with Alexa Fluor 647-conjugated phospho-STAT3 antibody (BD Biosciences) for 1 hour at RT.

### Immunohistochemistry

10µm thick frozen sections were fixed with acetone, and blocked with 1% BSA for 30 minutes at RT. For paraffin embedded tissue, the sections were fixed in 4% paraformaldehyde prior to 1% BSA blocking as noted above. Anti-human CD4 and CD8 FITC (green) conjugates or anti-human CD4, CD8, CD45, arginase-1, neutrophil lipocalin primary antibodies were incubated 1 hour and 4oC. For human CD45, arginase, and neutrophil lipocalin primary antibodies, Cy3 (red) conjugate antibody was used as secondary antibody in some cases. DAPI was used as the nuclear counterstain. Positive cells in 10 randomly selected fields at x40 magnification were quantitated. The microscope was Nikon, Eclipse E800, and the photos were captured with Nikon DS-Qi1Mc camera with NIS-Element AR 3.0 software.

### MDSC T-cell suppression assay

Human CD14+HLA-DR-/lo, CD15+HLA-DR-/lo and CD3+ cells were sorted from tumor bearing humanized mice spleen. Human CD3+ cells were co-cultured with CD14+HLA-DR-/lo and CD15+HLA-DR-/lo cells in complete RPMI media. After the cells were stimulated with 1mM/ml PMA and 1mg/ml Ionomycin and Golgistop for 4.5 hrs, harvested cells were performed membrane and intracellular cytokine staining with Cytofix/Cytoperm reagent kit (BD Biosciences). FACS data was collected on a FACSCalibur and was analyzed with FlowJo software (Tree Star). In some cases, stimulated T-cell proliferation was measured after 72 hours using 3H-thymidine incorporation. CD4+ T cells from humanized mice were isolated during the sorting of MDSC in parallel and activated using αCD3 and αCD28 (eBioscience) in some cases. MDSC or HLA-DR+ controls were co-cultured with autologous T cells at different (T cell:MDSC) ratios as noted in the figure legends. T-cell suppression assays were performed with 3% FCS in RPMI-1640.

### ELISA

Humanized mice were immunized with 2, 4-dinitrophenyl hapten-keyole limpet hemocyanin (DNP_23_-KLH, Biosearch Technologies) 0.1mg/mouse followed by a booster shot 3 weeks later. After 2 weeks the levels of DNP-specific humanized mice serum human IgG and its subclass antibodies were measured by enzyme-linked immunosorbent assay (ELISA, Life Technologies).

### Statistics

We used paired t-test to calculate two-tailed p values to estimate the statistical significance of differences between two treatment groups using Excel software. Error bars are standard error of means. P values are labeled in the figures. Kaplan-Meier curves were generated using GraphPad Prism software and analyzed with log-rank test.

## SUPPLEMENTARY MATERIALS FIGURES AND TABLES


