# Supplementary material for: Autologous reconstitution of human cancer and immune system in vivo
Source: Oncotarget. 2016 Dec 19;8(2):2053–68. doi: 10.18632/oncotarget.14026 (PMC5356779; doi:10.18632/oncotarget.14026)
Supplement: Supplementary file 1 [file oncotarget-08-2053-s001.pdf]

Autologous reconstitution of human cancer and immune system *in vivo*

Supplementary Material

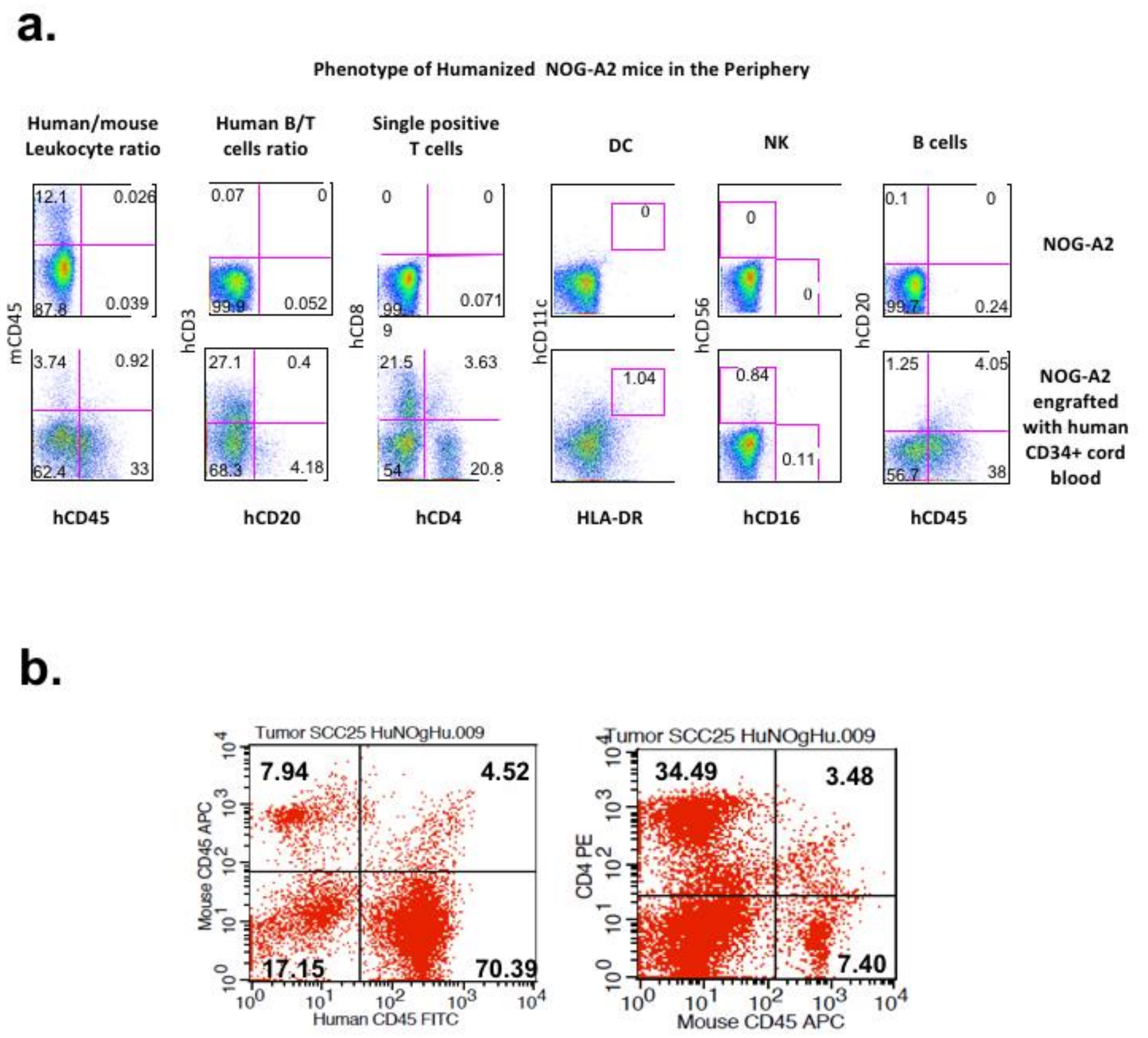

Supplemental Fig. 1. Allogeneic reconstitution of HLA-A2+ HNSCC tumor in humanized mice engrafted with HLA-A2+ cord blood. **a.** CD34+ cord blood cells from HLA-A2+ subjects were engrafted into NOG-A2 mice and their peripheral blood demonstrated human B-cells, single positive T-cells, dendritic cells, and NK cells. **b.** HLA-A2+ HNSCC cell line SCC25 (10^6) was

injected into the subcutaneous tissue, and once the tumor was over 10mm, the tumor infiltrating leukocytes segregated with Ficoll gradient and stained for both murine and human T-cells. Mononuclear TILs were stained for murine and human CD45 (left panel). Significant abundance of human CD4 were found in this ungated mononuclear population (right panel).

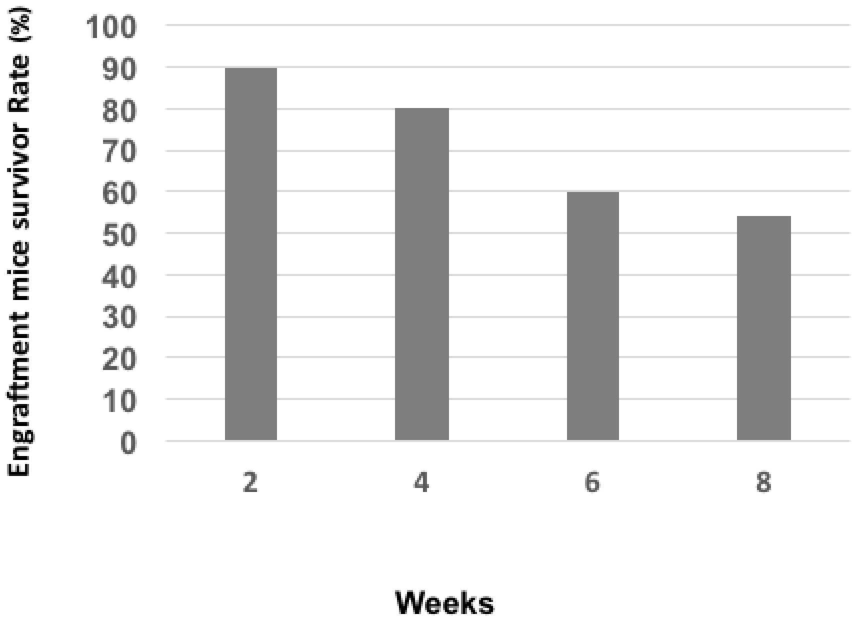

Supplemental Fig. 2. Depicted are the cumulative engraftment rates of CD34+ BMC. Approximately 110 mice were engrafted and their survival rates were noted till 8 weeks post adoptive transfer. One patient donor typically yielded 8-12 engrafted mice.

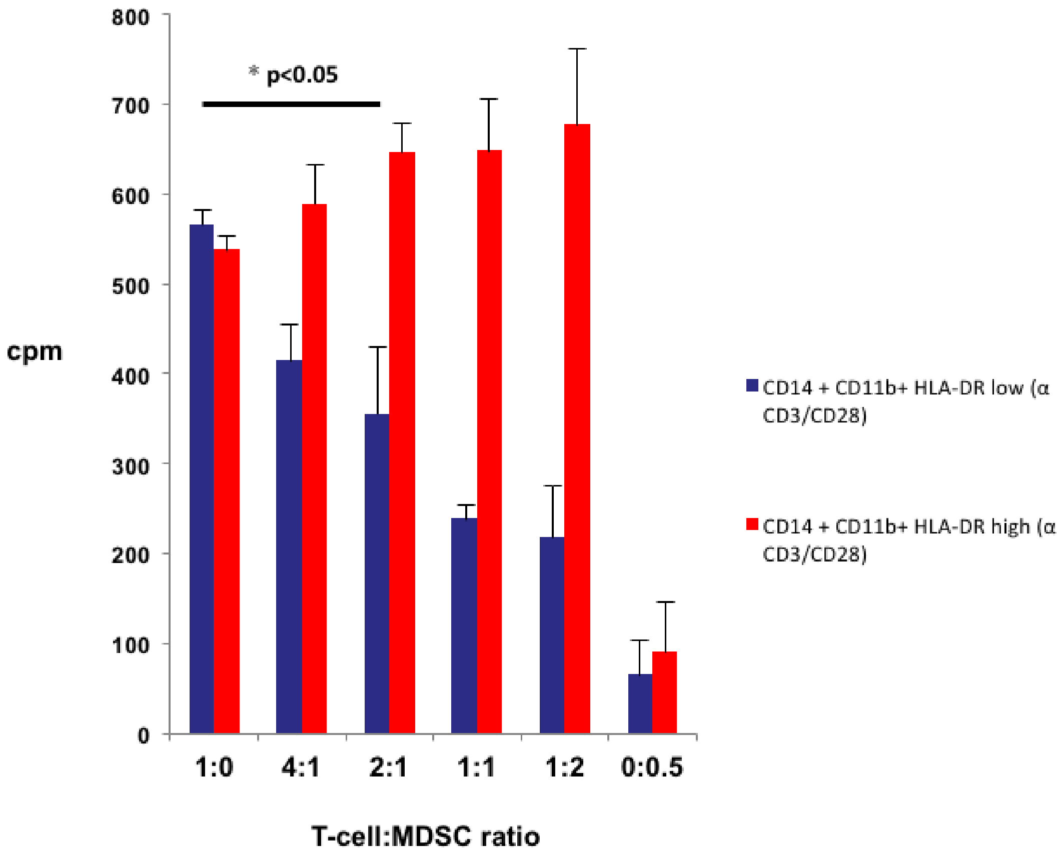

Supplemental Fig. 3. T-cell suppressive activity of MDSC sorted from tumor bearing humanized mice. CD14+DR^{low} and CD14^{+}DR^{high} cells from the spleen were sorted and mixed with human T-cells from normal subjects under T-cell stimulating conditions. T-cell and MDSC were mixed in ratios as indicated and ^{3}H-thymidine uptake of T-cells analyzed as measured in cpm. At 2:1 ratio of T-cells and MDSC, CD3/CD28 induced T-cell stimulation were suppressed at a significant level ($p<0.05$). Assays were performed in triplicates.
